# A comparative genomics and reductive dehalogenase gene transcription study of two chloroethene-respiring bacteria, *Dehalococcoides mccartyi* strains MB and 11a

**DOI:** 10.1038/srep15204

**Published:** 2015-11-06

**Authors:** Adrian Low, Zhiyong Shen, Dan Cheng, Matthew J. Rogers, Patrick K. H. Lee, Jianzhong He

**Affiliations:** 1 Department of Civil and Environmental Engineering, National University of Singapore, Singapore 117576; 2 B5423-AC1, School of Energy and Environment, City University of Hong Kong, Tat Chee Avenue, Kowloon, Hong Kong

## Abstract

Genomes of two trichloroethene (TCE)-respiring *Dehalococcoides* (*Dhc*) *mccartyi*, strains MB and 11a, were sequenced to identify reductive dehalogenases (RDase) responsible for oraganohalide respiration. Transcription analyses were conducted to verify the roles of RDase subunit A genes (*rdhA*) in chloroethene respiration. Some interesting features of the strain MB draft genome include a large genome size, two CRISPR-*cas* type I systems, and 38 *rdhA* genes. Strain 11a has a stream-lined genome with 11 *rdhA* genes, of which nine are distinct. Quantitative real-time PCR transcription analysis of RDase gene transcripts showed that a single RDase gene, designated *mbrA*, was up-regulated upon exposure to TCE and no other RDase genes were considerably expressed in strain MB. A single RDase gene, designated *vcrA*, was up-regulated upon exposure to TCE and expressed at a steady level until all chloroethenes were completely dechlorinated to ethene at 147 h in strain 11a. Overall, this study reports the genomes of two distinct *Dhc* strains; both contain numerous uncharacterized RDase genes, but in each strain only one such gene was expressed highly during organohalide respiration.

Organohalide-respiring *Dehalococcoides* (*Dhc*) *mccartyi* strains inhabit anaerobic environments and have environmentally relevant applications in remediating aquifers contaminated by organohalide compounds^1^. The enzyme responsible for catalyzing organohalide respiration is the reductive dehalogenase (RDase) which comprises a cobalamin centered catalytic subunit A (RdhA) and a membrane anchoring subunit B (RdhB)^2^,^3^. The *rdhA* genes of *Dhc* are an orthologous group, but the enzymes encoded by these genes have diverse organohalide substrates. For example, for the complete detoxification of tetrachloroethene (PCE) to ethene, *D. mccartyi* strain 195 (previously *D. ethenogenes* strain 195) utilizes PceA, which catalyzes PCE to trichloroethene (TCE) and TceA, which catalyzes stepwise dechlorination of TCE to *cis*-dichloroethene (DCE) and to vinyl chloride (VC)^2^,^4^,^5^. Other *Dhc* strains utilize different RDases for the dechlorination of the carcinogen VC to ethene, e.g. VcrA in *Dhc* strains 11a^6^, BTF08^7^,^8^, GT^9^, and VS^10^, and BvcA in *Dhc* strain BAV1^3^. Owing to their specificity, *rdhA* gene transcripts have been quantified as biomarkers of chloroethene dechlorination in contaminated environments^11^,^12^. To date, the complete genomes of *D. mccartyi* strains 195^13^, BAV1^14^, BTF08^7^, CBBD1^15^, DCMB5^7^, GT^9^, and VS^14^ as well as a phylogenetically close relative, *Dehalogenimonas lykanthroporepellens* strain BL-DC-9^16^, have been sequenced and made publicly available. The genomes of two other *D. mccartyi* strains (ANAS1 and ANAS2) were elucidated using whole-genome microarray specific to *Dhc*^17^. In addition to revealing new RDase genes, sequencing has also identified genes encoding proteins involved in the biosynthesis pathway of cyanocobalamin, which are essential cofactors for the function of the RDases, including proteins for scavenging cobalt/cobalamin^18^,^19^. Other interesting genomic features including clustered regularly interspaced short palindromic repeats (CRISPRs) and two high plasticity regions (HPRs) have also been identified^14^. Hence, elucidation of *Dhc* genomes, particularly strains with novel dechlorination activities can provide more insights into the biology of these organisms.

*D. mccartyi* strains MB and 11a were isolated from organohalide contaminated sediments in San Francisco, USA and Hubei, China, respectively^17^,^20^. A previous phylogenetic study of *Dhc* 16S rRNA genes showed differences at variable regions 2 and 6 that allowed for the assignment of three subgroups; namely Cornell, Pinellas and Victoria^21^. Phylogenetic alignment of the 16S rRNA gene places strain MB in the Cornell subgroup and strain 11a in the Pinellas subgroup (see Supplementary Fig. S1 online). However, subgroup affiliation cannot be used to predict the type of organohalide *Dhc* strain is capable of respiring. Strain MB only utilizes PCE/TCE metabolically^20^ while strain 11a can respire TCE, all DCE isomers, VC and 1,2-dichloroethane to ethene^6^. Strain MB is notable for producing *trans*-DCE as the predominant product from TCE respiration whereas the majority of *Dhc* strains produce *cis*-DCE as an intermediary product^22^. The only other *Dhc* with TCE respiratory characteristics similar to strain MB is strain CBDB1, which does not respire beyond *trans*-DCE and harbors a *rdhA* gene with 98% similarity to the *mbrA* gene of strain MB^23^. However, this apparent *mbrA* orthologue in strain CBDB1 remains experimentally uncharacterized^24^. Characterization of strain MB using whole-genome microarray based on the genome of strain 195 showed that 88% of the genes in strain 195 were orthologous to strain MB^20^. Ten putative RDase genes were identified in strain MB via microarray analysis^20^. An approach using degenerate primers for *rdhA* genes identified the sequences of seven *rdhA* genes, of which three (*dceA3, dceA4, dceA7*) were identical to sequences detected in the previous microarray study^23^. The *mbrA* gene was identified to be responsible for dechlorination of TCE to *trans*-DCE in strain MB based on a transcription study using quantitative real-time PCR (qPCR)^23^.

Strain 11a is interesting among VC-respiring *Dhc* strains because of its fast growth rate^6^. A previous study using whole-genome microarray based on the genomes of four *D. mccartyi* strains, 195, BAV1, CBDB1, and VS^17^, showed that strain 11a was most similar to strains BAV1 (89.5%) and CBDB1 (85.4%) at the genomic level. However, many genes in the HPRs of previously sequenced strains were absent, indicating that genes in HPRs are likely to be strain specific^6^. From the same microarray study, 11 putative *rdhA* genes were identified in strain 11a including the *vcrA* gene, which was expressed more than tenfold upon exposure to TCE, *trans*-DCE or VC, implicating its involvement in all steps of dechlorination^6^. However, the expression of the remaining 10 *rdhA* genes remains to be examined.

While previous studies have identified some *rdhA* genes in strains MB and 11a using microarray and PCR^6^,^20^, these methods were unable to detect novel sequences because of poor probe or primer specificity. Therefore, in this study, genomes of strains MB and 11a were sequenced, assembled and compared to the completed genomes of seven other *Dhc* strains to better understand genotypic characteristics. Furthermore, based on the *rdhA* sequences identified in the two strains, transcript expression studies were performed to determine the possible involvement of other RDase in the chloroethene dechlorination process.

## Results

### Genomic features of strains MB, 11a and comparisons with other *Dhc* strains

**Figure 1:**
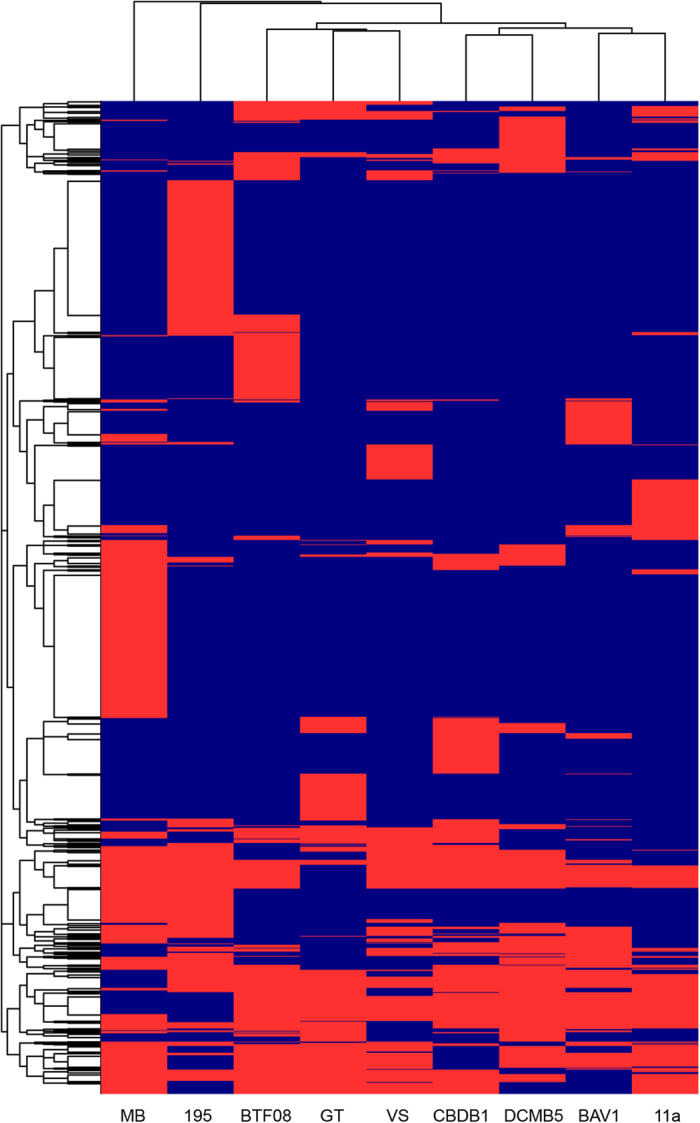
Heatmap and dendogram comparisons of non-core CDS of *D. mccartyi* strains 11a, 195, BTF08, BAV1, CBDB1, DCMB5, GT, MB, and VS. Red indicates presence and blue indicates absence. In total, 1,590 CDS were compared.

Draft genomes of strains MB and 11a comprise 11 and 6 scaffolds, respectively (see Supplementary Table S1 online), with total genome sizes of 1.58 Mb and 1.32 Mb, containing 1,575 and 1,346 protein coding sequences (CDS), and having a G + C content of 48.3% and 47.2%, respectively (Table 1). A comparison of orthologous CDS found in seven other *D. mccartyi* strains 195, BAV1, BTF08, CBDB1, DCMB5, GT, and VS, revealed that 61.8% (1029/1666) and 73.7% (1029/1397) of the genome in strains MB and 11a, respectively, were core genes (see Supplementary Data 1 online). Genes unique to strains MB and 11a comprise 11.0% (183/1,666) and 4.4% (62/1,397) of their genomes, respectively (see Supplementary Data 2 and 3 online). A combined dendrogram and heatmap of non-core genes among all seven *Dhc* strains show that strains MB and 195 of the Cornell subgroup share a higher number of orthologous genes with each other than with other *Dhc* strains. Strains 11a and BAV1 of the Pinellas subgroup share a higher number of orthologous genes with each other than with other *Dhc* strains (Fig. 1). Interestingly, strain VS, which belongs to the Victoria subgroup, is phylogenetically more similar to *Dhc* strains of the Pinellas subgroup at the genomic level (Fig. 1). This contrasts with 16S rRNA gene phylogenetic assignment of *Dhc* strains that places the Victoria subgroup closer to the Cornell subgroup (see Supplementary Fig. S1 online).

**Figure 2:**
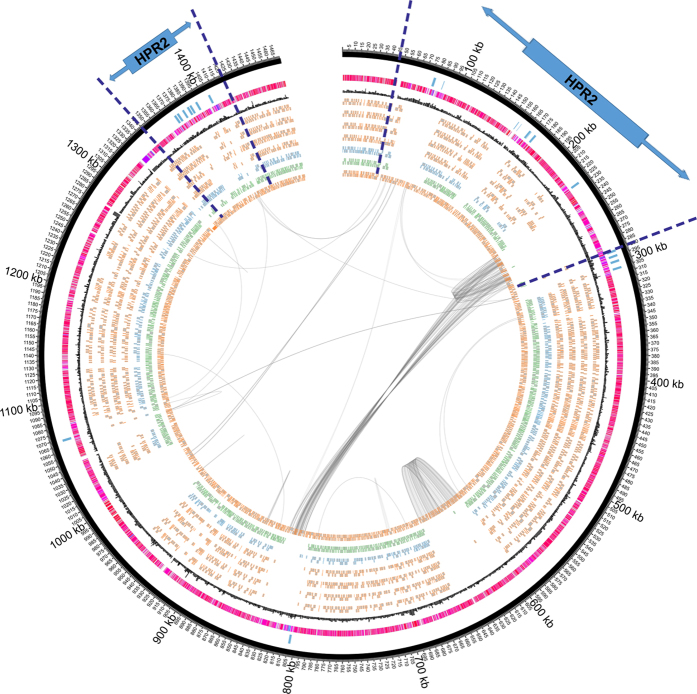
Circular map of *Dhc* genomes using strain 195 as the reference genome. Genomic coordinates are shown as 100 kbp intervals. From the outer ring, Ring 1 shows the *rdhAB* genes of strain 195. Ring 2 shows the G + C content of strain 195 shaded in red scale. Ring 3 shows a histogram of codon adaption index or codon usage bias of each gene larger than 200 codons, shaded in greyscale according to their CUB value for emphasis, black indicates highest CUB value of strain 195. Rings 4 to 9 are CDS orthologous to strain 195 in strains GT, CBDB1, VS, BAV1, 11a, and MB, respectively. Curved lines inside circles adjoin repeated elements. The two HPRs of strain 195 are marked with arrows and dashed lines.

**Figure 3:**
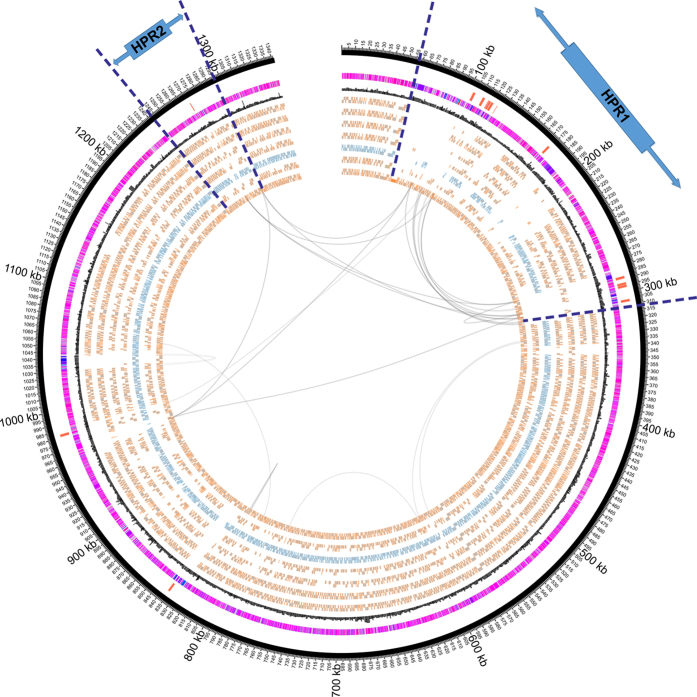
Circular map of *Dhc* genomes using strain BAV1 as the reference genome. Genomic coordinates are shown as 100 kbp intervals. From the outer ring, Ring 1 shows the *rdhAB* genes of strain BAV1. Ring 2 shows the G + C content of strain BAV1 shaded in red scale. Ring 3 shows a histogram of codon adaption index or codon usage bias of each gene larger than 200 codons, shaded in greyscale according to CUB value for emphasis, black indicates highest CUB value of strain BAV1. Rings 4 to 9 are CDS orthologous to strain BAV1 in strains GT, CBDB1, VS, 195, 11a, and MB, respectively. Curved lines inside circles adjoin repeated elements. The two HPRs of strain BAV1 are highlighted by arrows and dashed lines.

To identify the genes within the HPRs of strains MB and 11a, genomes were aligned to their respective closest genotypic relative, namely strains 195 and BAV1, respectively, as reference genomes. Because of the high variability among genes within the HPRs of *Dhc* strains, only those genes that adjoin the HPRs of reference genomes were identified to demarcate the HPRs in strains MB and 11a (Figs. 2 and 3). In strain MB, HPR-1 genomic position is from 58,100 to 231,199 and HPR-2 is from 1,137,418 to 1,349,625. In the reference genome of strain BAV1, HPR-1 genomic position is from 57,519 to 312,176 and HPR-2 is from 1,243,555 to 1,296,003^14^. The HPR-1 of strain MB is 148,981 bp long with 26.5% (45/170) of the CDS unique to strain MB, while HPR-2 is 323,466 bp with 82.1% (303/369) of the CDS unique to strain MB (Fig. 2 and see Supplementary Data 4 online). The HPR-1 of strain 11a is 80,313 bp with 27.1% (41/151) of the CDS unique to strain 11a, while HPR-2 is 39,896 bp long with no identifiable unique CDS (Fig. 3 and see Supplementary Data 5 online). Supplementary Data 4 and 5 (online) show the CDS within HPRs of strains MB and 11a, respectively.

### Identification of RDase genes in strain MB

Supplementary Data 6 and 7 (online) list the identified *rdhA* and *rdhB* gene sequences of strains MB and 11a, respectively. Thirty eight orthologous *rdhA* genes were identified in strain MB, of which four (DehaMB_1_0001, DehaMB_1_0161, DehaMB_1_0162, and DehaMB_8_0246) are partial sequences. Among the four RDase genes, DehaMB_1_0162 (1,148 bp) and DehaMB_8_0246 (146 bp) are incomplete sequences attributable to their locations at the ends of scaffolds 1 and 8. The sequences of DehaMB_1_0001 and DehaMB_1_0161 were found to be interrupted by a *rdhB* gene (DehaMB_1_0002) and a *rdhA* gene (DehaMB_1_0162). Three duplicated *rdhA* sequence pairs (DehaMB_1_0001 and DehaMB_5_0001, DehaMB_5_0157 and DehaMB_5_0005, DehaMB_5_0160 and DehaMB_8_0008) were found in the genome. However, it has to be noted that DehaMB_1_0001 is only identical to DehaMB_5_001 for the first 720 bp before it is disrupted by a *rdhB* gene (DehaMB_1_0002). Hence, 32 of the 38 *rdhA* genes are distinct full length sequences ranging from 1,391 to 1,715 bp (463 to 571 aa). While two genes (DehaMB_2_0594 and DehaMB_5_0010) are present in all nine *Dhc* strains compared in this study, five (DehaMB_1_0044, DehaMB_4_0168, DehaMB_5_0160, DehaMB_8_0008, and DehaMB_8_0011) are unique to strain MB.

In the strain MB genome, nine *rdhA* genes (DehaMB_1_0051, DehaMB_1_0053, DehaMB_1_0059, DehaMB_1_0119, DehaMB_1_0129, DehaMB_5_0001, DehaMB_5_0004, DehaMB_5_0010, and DehaMB_5_0160) do not have an accompanying *rdhB* gene. Three *rdhB* genes (DehaMB_2_0502_1, DehaMB_2_0506_1, and DehaMB_5_0156_1) were manually curated and amended with an additional number to differentiate them from neighbouring CDS annotated by codon prediction software. The failure of codon prediction software finding one of these CDS could be attributed to a rare start codon in DehaMB_2_0502_1 (GTG). This alternate start codon occurs in only about 11% of bacterial genomes sequenced^25^. However, it remains unknown why DehaMB_2_0506_1 was not annotated as a CDS despite having regular start and stop codons, ATG and TAA, respectively and DehaMB_5_0156_1 is a short 161 bp sequence truncated by a *rdhA* gene (DehaMB_5_0157) (see Supplementary Data 7 online). Failure to identify short genes is a known issue in several gene prediction software packages^26^. Three *rdhB* genes (DehaMB_5_0159, DehaMB_8_0004, and DehaMB_8_0007) with low homologies (59% amino acid similarity) to available sequences in public databases were found (see Supplementary Data 7 online). Supplementary Fig. S2 and S3 (online) show phylogenetic trees of full length RdhA and RdhB found in strains MB and 11a along with the two top hits according to BLAST searches based on deduced amino acid sequences, respectively. The *mbrA* gene is located adjacent to a *rdhD* gene (DehaMB_2_0504) that encodes a putative DNA binding response regulator and a *rdhC* gene (DehaMB_2_0505) that encodes a histidine kinase sensor (see Supplementary Data 8 online).

### Identification of RDase genes in strain 11a

Eleven *rdhA* genes were identified in strain 11a, of which Deha11a_2_0134 (281 bp) and Deha11a_6_0020 (542 bp) are truncated sequences, interrupted by a *rdhA* gene (Deha11a_2_0135) and a *rdhB* gene (Deha11a_6_0019), respectively. All nine full-length *rdhA* genes have an accompanying *rdhB* gene. Deha11a_6_0020 is located adjacent to a transposase gene (Deha11a_6_0021). However, only a single full length *rdhA* gene (Deha11a_3_0062) and an accompanying *rdhB* (Deha11a_3_0063) were found in HPR-1 (see Supplementary Data 5 online). Homology of the identified putative *rdhA* genes was assessed by amino acid sequence comparison. Eight of the nine RdhA have high homologies (≥99% amino acid similarities) to strain BTF08, while one (Deha11a_2_0144) is most similar to (amino acid similarity 96%) to an RdhA in strains CBDB1 and DCMB5 (see Supplementary Data 6 online). The *vcrABC* operon consists of Deha11a_4_0536 (known herein as *vcrA*) adjacent to a *vcrB* gene (Deha11a_4_0534) and a *vcrC* (Deha11a_4_0534) gene. The *vcrABC* operon is located with two other *rdhAB* genes (Deha11a_4_0537 and Deha11a_4_0538) and a MarR transcriptional regulator (Deha11a_4_0539) (see Supplementary Data 9 online).

### Transcription of *rdhA* genes during dechlorination.

**Figure 4:**
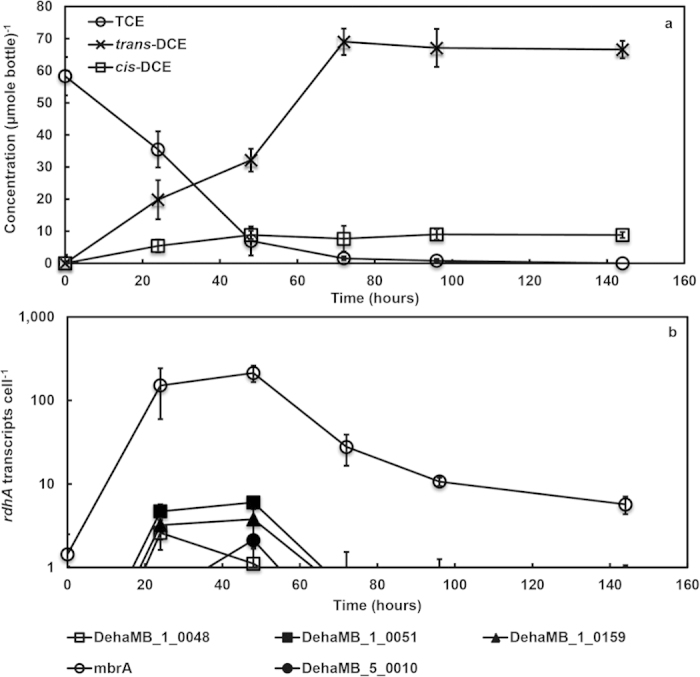
(**a**) Dechlorination profile of TCE and (**b**) transcript expression of *rdhA* genes in strain MB. The *mbrA* transcript was up-regulated 100 fold at 24 h and remained at a similar level after 48 h and decreased tenfold at 72 h corresponding with near complete dechlorination of TCE to *trans*-DCE. DehaMB_1_0048, DehaMB_1_0051 and DehaMB_1_0159 transcripts were up-regulated at 24 h while DehaMB_5_0010 transcript was expressed at 48 h. However, the transcripts of all four genes were expressed ≥53 fold less than the *mbrA* transcript at the highest level of expression. All *rdhA* transcripts, except *mbrA*, are expressed at less than 1 transcript cell^−1^ at 72 h. The remaining 27 *rdhA* genes had expression levels <1.0 transcript cell^−1^ and are not shown. Error bars, which may be smaller than the symbols, are averages of biological triplicates.

The transcription analysis of strain MB was performed using primers designed for the 32 distinct full-length *rdhA* genes. During TCE dechlorination to *trans*- and *cis*-DCEs (Fig. 4), the DehaMB_1_0048, DehaMB_1_0051, DehaMB_1_0159 and *mbrA* genes were up-regulated at 24 h, but *mbrA* transcripts were at least 58-fold more than the other three transcripts (Fig. 4b). At 48 h, DehaMB_5_0010 gene was expressed but was 100-fold less than the *mbrA* gene. The DehaMB_1_0051 and DehaMB_1_0159 genes remained at relatively the same level, whereas of DehaMB_1_0048 decreased by one-fold (Fig. 4b). After 72 h, only the *mbrA* transcript was still measurable, at 27 transcripts cell^−1^, while all other *rdhA* genes were less than 1 copy cell^−1^. The remaining 27 *rdhA* transcripts had less than 1 copy cell^−1^ at all timepoints (data not shown).

**Figure 5:**
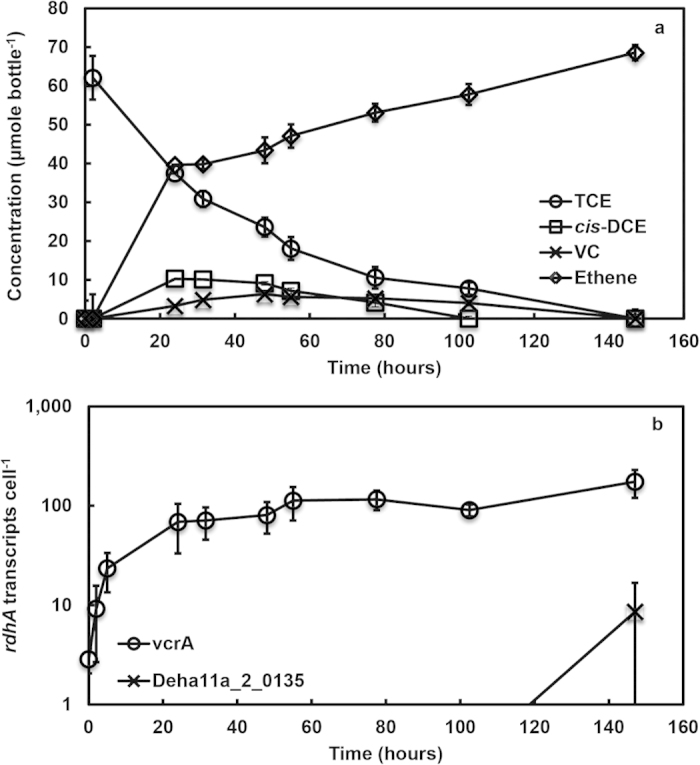
**(a)** Dechlorination profile of TCE and (**b**) transcript expression of *vcrA* and Deha11a_2_0135 genes in strain 11a. The *vcrA* transcript was expressed 3.2 fold at 9 h and continued to be expressed another 7.5 fold higher after 24 h, at which TCE was dechlorinated to *cis*-DCE and VC. The *vcrA* transcript remained high after complete dechlorination of chloroethenes to ethene after 147 h. Another *rdhA* transcript (Deha11a_2_0135) showed quantifiable expression at 147 h but only in a single culture out of triplicates. The remaining seven *rdhA* genes had expression level <1.0 and are not shown. Error bars which may be smaller than the symbols are averages of biological triplicates.

In strain 11a, out of the nine full-length *rdhA* genes, only *vcrA* was expressed during the dechlorination of TCE to DCEs (Fig. 5). *VcrA* gene transcripts increased nearly an order of magnitude within 9 h of exposure to TCE and continued to increase for 24 h, remaining stable after which all chloroethenes were completely transformed to ethene after 147 h (Fig. 5). While there was apparently a nearly tenfold increase in Deha11a_2_0135 transcripts at 146 h, this can likely be attributed to experimental variability as the apparent increase occurred in only one out of three cultures (Fig. 5b). Transcription experiments of strain 11a cultures exposed to only 1,1-DCE, *cis*-DCE or VC (see Supplementary Fig. S4 online) confirmed the expression of *vcrA* gene during DCE and VC dechlorination. This contrasted with the expression of the other *rdhA* genes that were either below measurable limits or showed no difference with the controls in the absence of chloroethenes (data not shown). There was no loss of TCE in the sterile controls for both strains MB and 11a (see Supplementary Fig. S5 online).

### Identification of CRISPR and CRISPR associated (*cas*) genes.

Strain MB has two conserved repeated sequences of 5′-ATTGCTCCCCACGCGGG-AGCATGAATTGAAAC-3′ from genome position 125,157 to 126,575 and 5′-GTCGCT-CCCCGTGCGGGAGCGTGAATTGAAAC-3′ from genome position 503,324 to 506,386, of which each repeat sequence is followed by 21 bp and 46 bp spacers (a unique sequence), respectively (see Supplementary Data 8 online). Two nearly identical modules of Type I CRISPR-*cas* genes (DehaMB_1_0110 to 1_0115 and DehaMB_2_0512 to 2_0518) are present in the genome of strain MB as identified by the presence of *cas1* (DehaMB_1_0114 and DehaMB_2_0517), *cas2* (DehaMB_1_0115 and DehaMB_2_0518) and *cas3* (DehaMB_1_0110 and DehaMB_2_0512) genes that encode for nucleases (see Supplementary Data 8 online). A *cas4* (DehaMB_2_0516) gene encoding a RecB-like nuclease was identified in one set of CRISPR associated genes, located within HPR-1, suggesting horizontal acquisition. No CRISPR-*cas* gene was found in strain 11a.

## Discussion

*Dhc* strains are important to the bioremediation of environment contaminated with various halogenated compounds, but are difficult to characterize because of challenges in culturing them. In order to gain more genetic information on *Dhc*, the genomes of two previously isolated strains, MB^20^ and 11a^6^, were sequenced and analyzed in this study. Whole genome comparison of *Dhc* strains shows close genomic similarities between strains MB and 195 as well as between strains BAV1 and 11a, which are in congruence with the findings from whole genome microarray studies of the two strains^6^,^20^. Surprisingly, *de novo* sequencing did not reveal any previously unidentified *rdhA* genes in strain 11a^6^. This study shows that strain 11a has the same number of RDase as strain BAV1 (see Supplementary Data 1 online). Interestingly, strains 11a and BAV1 are closest phylogenetically and genomically than all the other *Dhc* strains.

Despite the presence of multiple full-length *rdhA* genes (32) in strain MB, transcription analysis confirmed that the *mbrA* gene transcript was expressed the highest comparing with six other *rdhA* genes, including DehaMB_1_0159 and DehaMB_5_0010 gene transcripts. These six *rdhA* genes were expressed ≥70 fold less than *mbrA* gene transcripts, which is consistent with a previous study^23^. The lack of a RDase with close homology to the more common TceA suggests that *cis*-DCE, produced as a minor product of TCE dechlorination by strain MB, is most likely a product of secondary activity by MbrA rather than a product of a specific RDase. Minor products of dechlorination have been demonstrated in an enzymatic study of TceA, which produces *trans*-DCE as a secondary product and *cis*-DCE as a primary product of TCE dechlorination^2^. The remaining 27 *rdhA* genes quantified had transcript expression much less than those of *mbrA* gene transcripts, especially during TCE dechlorination by strain MB (data not shown). Nevertheless, the role of another RDase cannot be ruled out until the complete genome of MB is closed and other possible RDase genes are investigated further.

Although no new RDase was identified in strain 11a when compared to the previous microarray study^6^, genome sequencing enabled primers to be designed for the transcription study. The transcriptional analysis confirmed VcrA as the sole enzyme responsible for TCE, DCEs and VC dechlorination. While the previous study of strain 11a implicated VcrA in the dechlorination of those chloroethenes, the involvement of other RDases had not been previously discounted^6^. Interestingly, despite the identical (100% amino acid similarity, 97% nucleotide similarity) VcrA sequences in strains 11a, VS and GT, strain 11a had a relatively higher dechlorination rate (258 nmol Cl^−1^ min^−1^ mg of protein^−1^ in strain 11a; 56 nmol Cl^−1^ min^−1^ mg of protein^−1^ in strain VS; 92 nmol Cl^−1^ min^−1^ mg of protein^−1^ in strain GT) than any known VC-respiring *Dhc* strains, suggesting the contribution of other physiological factors^6^. However, closing the genome of strain 11a would be necessary to rule out the presence and involvement of other RDases in chloroethene respiration.

Comparison of the number of genes unique to the genomes of strains MB and 11a suggests that the former has a higher diversity of functional genes. It should be noted that a large proportion (47.5% for strain MB and 74.1% for strain 11a) of the unique genes are uncharacterized proteins (see Supplementary Data 4 and 5 online). Several of the unique genes in strain MB have annotations to proteins found in Archaea, such as ABC transporter proteins, cation chelating proteins and periplasmic binding proteins (see Supplementary Data 4 online). In general, such proteins have functions relating to binding and transport of cofactors, such as iron and cobalt, into the cell for enzymatic function. For example, iron is an essential cofactor for nitrogenase enzyme in nitrogen fixation^27^, and Hym-type iron hydrogenases that are essential enzymes in *Dhc* strains^28^. Phylogenetic analysis of the five RDases unique to strain MB showed low homologies (41% to 75% amino acid similarities) to available sequences in the public databases (see Supplementary Data 6 online). This suggests that strain MB might be capable of respiring a wider range of organohalides not previously characterised.

Strain MB has a Type I CRISPR-*cas* system as identified by the *cas3* gene^29^. However, the *cas6* or *cas7* endoribonuclease genes responsible for CRISPR RNA genesis is absent, which suggests an incomplete CRISPR-*cas* system or involvement of an uncharacterised gene in CRISPR RNA genesis^30^. Interestingly, annotation of the *cas* genes showed nucleotide similarities of 44% to 62% to those genes found in *Geobacter*, *Desulfotomaculum* and an unknown sulphur-oxidising bacterium (see Supplementary Data 8 online). Incidentally, these taxa are phylogenetically distant to *Dhc*, suggesting that these genes might be horizontally acquired from distantly related bacteria. Another interesting feature of the strain MB genome is that it contains 14 more phage associated integrases than strain 11a. Given the significance of horizontally acquired genes in *Dhc*, particularly with RDase genes^31^, CRISPR-*cas* genes may suggest mechanisms for *Dhc* to defend against phage so as to maintain genome integrity and even to regulate the expression of transcripts that might not be beneficial to the host^30^. Searches on the CRISPRdb database^32^ revealed Type I CRISPR-*cas* genes in strains CBDB1^33^ and GT with the former lacking a *cas2* gene and the latter having an additional *cas5* gene^31^,^34^. Only strain DCMB5 has two near identical modules of Type I CRISPR-*cas* system similar to strain MB, but with additional *cas5, cas6* and *cas7* genes^32^. Interestingly, strain BAV1 has just a single CRISPR sequence and a spacer at genome positions 283,847 to 283,946 but no *cas* genes, suggesting an incomplete phage defence^32^. *Dhc* without CRISPR-*cas* genes include strains 11a, 195, BTF08, VS, and the phylogenetically closest genus *Dehalogenimonas lykanthroporepellens* strain BL-DC-9^16^,^32^. It remains to be tested if *Dhc* strains harbouring CRISPR-*cas* genes are evolutionarily better equipped to adapt in the environment against transduction than those that do not.

Four unique genes are likely to be involved in cyanocobalamin synthesis pathway in strain MB. These include a cyanocobalamin importer permease protein BtuC (DehaMB_1_0081) responsible for the transmembrane transport of cyanocobalamin^35^, a cobalt chelatase CobN (DehaMB_1_0066) that is part of the CobNST complex involved in the insertion of cobalt onto the corrin ring^36^, CbiET (DehaMB_1_0073) responsible for the methylation of cobalt-precorrin-6 intermediates^36^, and an undefined cobalamin synthesis protein of the P47K family (DehaMB_1_0090). The presence of these cyanocobalamin associated genes suggests that *Dhc* have more functional proteins involved in the cobalamin synthesis pathway than previously thought^37^. Thus far, only strain 195 is known to harbor two sets of identical genes (DET0657–0659/DET0691–0693) responsible for synthesis of the lower ligand base to the cobryic acid structure of cyanocobalamin^37^, suggesting that scavenging of cobalamin is still the predominant survival strategy for maintaining a functional RDase in strain MB. The recent elucidation of the crystal structure of PceA from *Sulfurospirillum multivorans* revealed a pocket within the PceA structure that contains a cyanocobalamin, stressing the importance of the co-factor in dechlorination^18^. The CDS of strains MB and 11a associated with the cobalamin synthesis pathway are listed in Supplementary Data 11 and 12 (online). Another gene unique to strain MB is a rubrerythrin (DehaMB_1_0097) that could confer protection against oxidative stress^38^. Other unique genes are five CDS that are closest to *D. lykanthroporepellens* strain BL-DC-9, of which only one has an annotated function. This gene encodes a radical S-adenosyl-L-methionine (SAM) domain protein that generates radical species by reductive cleavage of SAM, which is required in many biochemical pathways, including cyanocobalamin synthesis^39^.

There are far fewer unique CDS in the genome of strain 11a. Among the unique CDS is a beta-lactam domain protein (Deha11a_3_0143) that may function to hydrolyse beta-lactam antibiotics^40^ or bind to nucleic acids in DNA repair (Deha11a_3_0143)^41^, a DEAD/DEAH box RNA helicase (Deha11a_3_0142)^42^, and a HigA (Deha11a_5_0002) anti-toxin protein that is part of a type I toxin/anti-toxin (TA) system^43^ (see Supplementary Data 9 online). A second TA locus, which encodes for a type II TA system, RelE (Deha11a_4_0255) and RelB (Deha11a_4_0256) was found in the genomes of strains 11a, MB and BAV1. TA systems have been postulated to increase survivability in times of cellular stress, such as in the presence of antimicrobials or possibly, from saturated organohalides^43^, however their significance in *Dhc* has not been shown. While strain 11a shares several similar RdhA with strain BTF08, PceA and TceA are not among them. The *vcrABC* operon of strain 11a is adjacent to a cluster of putative integrative genes (Deha11a_4_0524 to Deha11a_4_0532) that comprise a *ssrA-*specific genomic island, similar to other *vcrABC* harbouring *Dhc* strains. A notable exception is the *vcrABC* operon of strain GT that is located in a second genomic island downstream of an *ssrA*^31^. Given the global distribution of VC-respiring strains, there appears to be a time in which these *vcrA* genes were horizontally inserted into *Dhc*, however the vector for this insertion has not been discovered^31^.

One of the obvious genomic features unique to strain MB is its large genome size (1.58 Mb), which is bigger than that of strain 195 (1.47 Mb) and potentially as big as the genome of *D. lykanthroporepellens* (1.69 Mb), a close relative of *Dhc* (see Supplementary Data 1 online). Even the HPRs, especially HPR-2 (323,466 bp), are larger than the largest previously reported *Dhc* HPR in strain DCMB5 (HPR-1: 286,952 bp). The HPRs in *Dhc* genomes characteristically contain a large number of *rdhA* orthologues^14^. Twenty three *rdhA* genes, including the *mbrA* gene, were found in both HPRs of strain MB.

Strain 11a has a genome similar to those of other *Dhc* strains of the Pinellas subgroup, especially to strain BAV1 including the HPRs (1.34 Mb). This is surprising considering that these two strains were isolated from different parts of the world and would have presumably encountered different selective pressures for genes in the HPRs^44^. There is nothing obvious in the genome that suggests a mechanism for the rapid growth exhibited by strain 11a. A recent paper showed that a MarR protein from strain CBDB1 could act as a transcriptional repressor when induced in *E. coli* strain LS5 that carried a vector (pBAD30) constructed with the MarR gene from CBDB1^45^. Given that there is a MarR gene adjacent to the *vcrABC* operon, differences in the regulatory mechanisms of different MarR proteins may be present.

In conclusion, we have identified the RDases of two chloroethene-respiring *Dhc* strains. Strain MB has 32 different RDase but just one, *mbrA*, was expressed to dechlorinate TCE in a stepwise manner to predominantly *trans*-DCE. Strain 11a has nine different RDases, but just one, *vcrA*, was expressed to dechlorinate TCE to ethene in a stepwise fashion. The large pool of RDases suggests that both strains have the potential to respire other organohalides. The presence of two CRISPR-*cas* genes in strain MB suggests a mechanism for phage defence and gene regulation. When both genomes are compared with other sequenced strains of *Dhc*, strain MB has a genome most similar to strain 195 but has twice the number of *rdhA* genes than strain 195. While strain 11a has a genome most similar to strain BAV1, the *rdhA* genes are most similar to those of strain BTF08.

## Methods

### Chemicals

All chemicals (>97% purity) were purchased from Sigma-Aldrich (St. Louis, MO, USA).

### Bacterial Culturing and Analytical Methods

Strains MB and 11a were cultured in defined minimal salts medium under anaerobic conditions as previously described^6^,^20^. Briefly, 100 mL of anaerobic medium was stored in 160 mL serum bottles capped with rubber stoppers and sealed using aluminium crimp caps. The headspace was flushed and maintained with nitrogen and carbon dioxide (5:1). Cultures were amended with H_2_ (500,000 ppm) and acetate (5 mM) and incubated in the dark at 30 °C without agitation. To quantify chloroethene and ethene concentrations, 100 μL of headspace from each culture was injected using a 250 μL syringe with luer-lock (Hamilton, Nevada, USA) into an Agilent gas chromatography 6890N equipped with a flame ionization detector and a GS-GasPro column (Agilent Technologies, Santa Clara, CA, USA) using a method as previously described^46^. Nominal concentration of organohalides and ethene are expressed as μmol per bottle (160 mL).

### Nucleic acid extraction, Quantitative PCR, and Transcription Experiments

DNA used for genome sequencing was extracted from cells concentrated from 1 L of strains MB or 11a cultures by centrifugation at 8000 × g for 20 mins in 50 mL conical tubes. High molecular weight genomic DNA was extracted using a Genomic-tip 100G kit (Qiagen GmbH, Hilden, Germany) as described in the manufacturer’s protocol. For the *rdhA* transcript expression study, RNA was extracted from 1.5 mL of cultures using the RNeasy mini kit (Qiagen GmbH, Hilden, Germany) according to the manufacturer’s protocol with some modifications, including a DNA digestion step, as previously described^6^. A detailed description of the RNA extraction methodology can be found as Supplementary Methods online. DNA was extracted from 1 ml of culture using the Qiagen Blood and Tissue kit (Hilden, Germany) according to the manufacturer’s protocol with slight modifications, as previously described^23^.

Total RNA was converted to cDNA using a Sensiscript reverse transcription kit (Qiagen GmbH, Hilden, Germany) with random hexamers (Promega, Madison, WI, USA) as described in the manufacturer’s protocol. Information on qPCR conditions and primers used can be found as Supplementary Methods and Supplementary Data 10 online.

Transcriptional analysis was conducted to identify functional and expressed *rdhA* genes among the identified RDase orthologues in strains MB and 11a. MB and 11a cultures were inoculated at 5% and 10% v/v, respectively, in 160 ml serum bottles, grown to completion (e.g., complete transformation of chlorinated substrate amendment to ethene in strain 11a and to *trans*-DCE and *cis*-DCE in strain MB) and then starved for 120 hours to minimize carryover of transcripts produced during initial cell growth. After starvation, biological triplicate parallel cultures were spiked with ~60 μM (nominal concentration) TCE, *cis*-DCE, 1,1-DCE or VC as indicated. Cells for extraction of total RNA (1.5 mL cells) and DNA (1.0 mL cells) were harvested from cultures at defined time points. DNA and RNA were extracted from cells as described. Cells were harvested regularly until all organohalides had been completely dechlorinated to characteristic endpoints. Negative controls for each condition were prepared without cell inocula and both with and without chlorinated substrate amendment.

### Genome Sequencing and Annotation

The genomes of MB and 11a were sequenced on an Illumina GA-II genome analyzer at the Beijing Genome Institute (BGI-Hong Kong). A paired-end sequencing method of 90 bp long reads in 200 bp insert library was used, yielding coverage depth of about 110 × per genome. The National Center for Bio-technology Information (NCBI) accession numbers for strains MB and 11a are stated in Table 1. Quality sequences were obtained using a threshold of 20 as quality score^47^. Detailed methodology on genome construction, open reading frame predictions, annotation, genome comparison, phylogenetic tree construction, heatmap of non-core genes and Clustered regularly interspaced short palindromic repeats (CRISPR) searches can be found as Supplementary Methods online. Adobe Illustrator CS6 was used to enhance the resolution of figures throughout this manuscript.

## Additional Information

**How to cite this article**: Low, A. *et al.* A comparative genomics and reductive dehalogenase gene transcription study of two chloroethene-respiring bacteria, *Dehalococcoides mccartyi* strains MB and 11a. *Sci. Rep.* **5**, 15204; doi: 10.1038/srep15204 (2015).

## Supplementary Material

Supplementary Information

Supplementary Data 1

Supplementary Data 2

Supplementary Data 3

Supplementary Data 4

Supplementary Data 5

Supplementary Data 6

Supplementary Data 7

Supplementary Data 8

Supplementary Data 9

Supplementary Data 10

Supplementary Data 11

Supplementary Data 12

## Figures and Tables

**Table 1:** Genome properties of *D. mccartyi* strains MB and 11a.

Parameter	MB	11a
GenBank ID	JGYD00000000	JGVX00000000
Tentative genome sizes (bp)	1,582,192	1,324,528
DNA G+C content (%)	48.3	47.2
Total genes	1,666	1,397
SSU rRNA genes	1	1
LSU rRNA genes	1	1
tRNA genes	46	46
Total protein coding genes	1,575	1,346
Core genes	1,029	1,029
% of core genes (Core genes/Total genes)	61.8	73.7
Non-core genes	637	368
Unique genes	183	62
% of unique genes (Unique genes/Total genes)	11.0	4.4
Transposases	5	9
Integrases	14	1
rdhA	38^a^	11^b^
rdhB	30^c^	9
MarR	34	9
Cobalamin synthesis associated genes	14	6
Cobalamin transport genes	10	3
CRISPR-*cas* repeats	2	0

^a^2 truncated and 2 partially sequenced genes.

^b^2 truncated genes.

^c^1 truncated gene.
