# Author Correction: A comparative genomics and reductive dehalogenase gene transcription study of two chloroethene-respiring bacteria, *Dehalococcoides mccartyi* strains MB and 11a

**DOI:** 10.1038/s41598-018-30575-7

**Published:** 2018-08-17

**Authors:** Adrian Low, Zhiyong Shen, Dan Cheng, Matthew J. Rogers, Patrick K. H. Lee, Jianzhong He

**Affiliations:** 1 Department of Civil and Environmental Engineering, National University of Singapore, Singapore, 117576 Singapore; 2 B5423-AC1, School of Energy and Environment, City University of Hong Kong, Tat Chee Avenue, Kowloon Hong Kong

Correction to: *Scientific Reports* 10.1038/srep15204, published online 06 November 2015

The original version of this Article contained an error where Supplementary Data 10 was a duplication of Supplementary Data 11. This error has now been corrected in the Supplementary Data that accompanies this Article.

