# Supplementary material for: A comparative genomics and reductive dehalogenase gene transcription study of two chloroethene-respiring bacteria, Dehalococcoides mccartyi strains MB and 11a
Source: Sci Rep. 2015 Nov 6;5:15204. doi: 10.1038/srep15204 (PMC4635342; doi:10.1038/srep15204)
Supplement: Supplementary Information [file srep15204-s1.pdf]

A comparative genomics and reductive dehalogenase gene transcription study of two chloroethene-respiring bacteria, *Dehalococcoides mccartyi* strains MB and 11a

Adrian Low, Zhiyong Shen, Cheng Dan, Matthew J. Rogers, Patrick K. H. Lee and Jianzhong He

Supplementary Figures

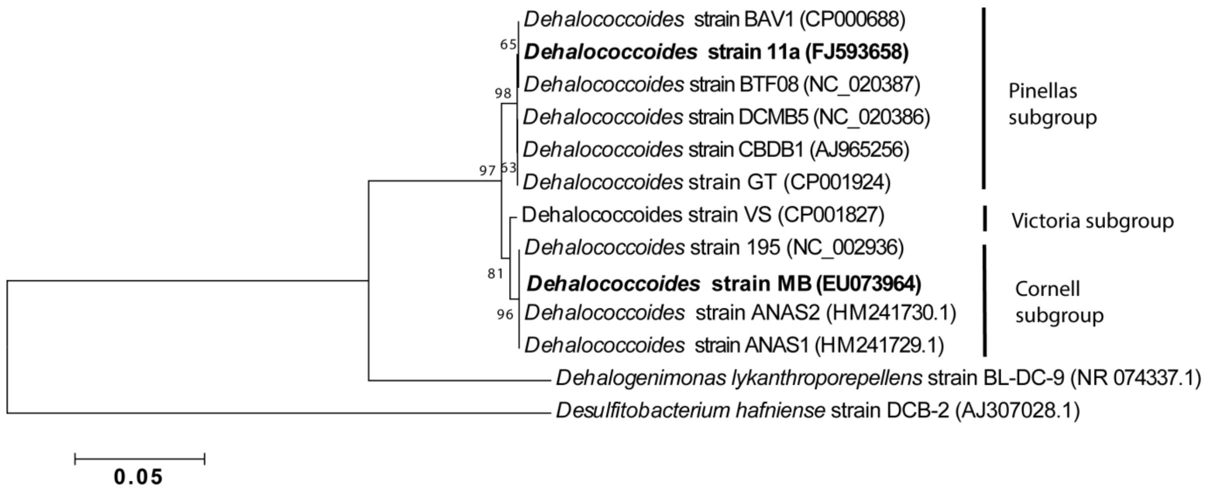

Supplementary Fig. S1. Neighbour joining tree of 16S rRNA genes of *Dhc* strains, numbers at nodes show bootstrap values using 1,000 replicates. Subgroup classification is indicated on the right. Scale bar shows 5% nucleotide base difference.

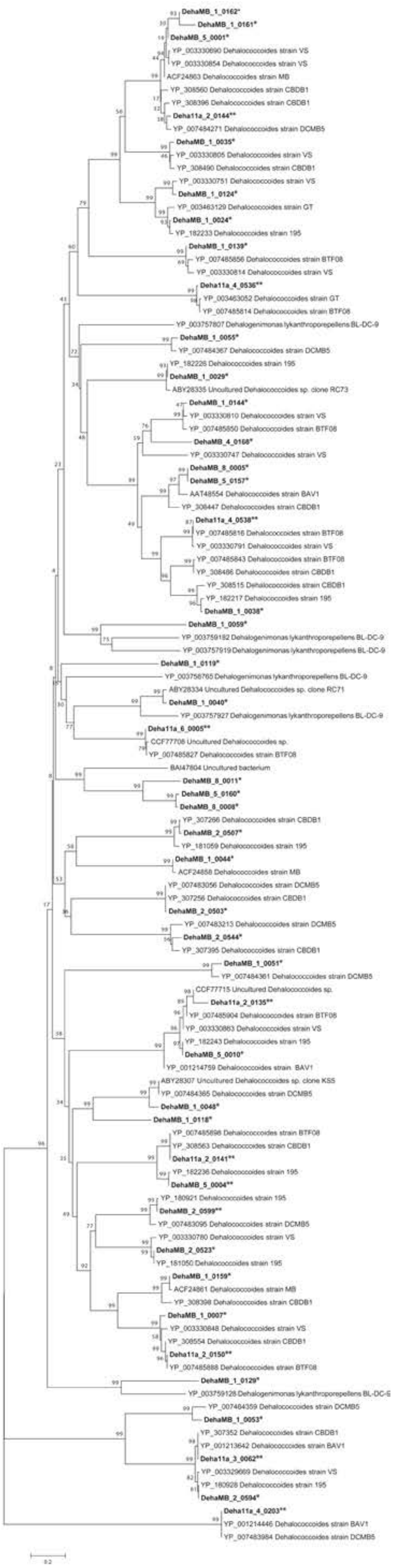
DehaMB_1_0162*
DehaMB_1_0161*
DehaMB_5_0001*
YP_003330690 Dehalococcoides strain VS
YP_003330854 Dehalococcoides strain VS
ACF24863 Dehalococcoides strain MB
YP_308560 Dehalococcoides strain CBDB1
YP_308396 Dehalococcoides strain CBDB1
Deha11a_2_0144**
YP_007484271 Dehalococcoides strain DCMB5
DehaMB_1_0035*
YP_003330805 Dehalococcoides strain VS
YP_308490 Dehalococcoides strain CBDB1
YP_003330751 Dehalococcoides strain VS
DehaMB_1_0124*
YP_003463129 Dehalococcoides strain GT
DehaMB_1_0024*
YP_182233 Dehalococcoides strain 195
DehaMB_1_0139*
YP_007485856 Dehalococcoides strain BTF08
YP_003330814 Dehalococcoides strain VS
Deha11a_4_0536**
YP_003463052 Dehalococcoides strain GT
YP_007485814 Dehalococcoides strain BTF08
YP_003757807 Dehalogenimonas lykanthroporepellens BL-DC-9
DehaMB_1_0055*
YP_007484367 Dehalococcoides strain DCMB5
YP_182226 Dehalococcoides strain 195
DehaMB_1_0029*
ABY28335 Uncultured Dehalococcoides sp. clone RC73
DehaMB_1_0144*
YP_003330810 Dehalococcoides strain VS
YP_007485850 Dehalococcoides strain BTF08
DehaMB_4_0168*
YP_003330747 Dehalococcoides strain VS
DehaMB_8_0005*
DehaMB_5_0157*
AAT48554 Dehalococcoides strain BAV1
YP_308447 Dehalococcoides strain CBDB1
Deha11a_4_0538**
YP_007485816 Dehalococcoides strain BTF08
YP_003330791 Dehalococcoides strain VS
YP_007485843 Dehalococcoides strain BTF08
YP_308486 Dehalococcoides strain CBDB1
YP_308515 Dehalococcoides strain CBDB1
YP_182217 Dehalococcoides strain 195
DehaMB_1_0038*
DehaMB_1_0059*
YP_003759182 Dehalogenimonas lykanthroporepellens BL-DC-9
YP_003757919 Dehalogenimonas lykanthroporepellens BL-DC-9
DehaMB_1_0119*
YP_003758765 Dehalogenimonas lykanthroporepellens BL-DC-9
ABY28334 Uncultured Dehalococcoides sp. clone RC71
DehaMB_1_0040*
YP_003757927 Dehalogenimonas lykanthroporepellens BL-DC-9
Deha11a_6_0005**
CCF77708 Uncultured Dehalococcoides sp.
YP_007485827 Dehalococcoides strain BTF08
BAI47804 Uncultured bacterium
DehaMB_8_0011*
DehaMB_5_0160*
DehaMB_8_0008*
YP_307266 Dehalococcoides strain CBDB1
DehaMB_2_0507*
YP_181059 Dehalococcoides strain 195
DehaMB_1_0044*
ACF24858 Dehalococcoides strain MB
YP_007483056 Dehalococcoides strain DCMB5
YP_307256 Dehalococcoides strain CBDB1
DehaMB_2_0503*
YP_007483213 Dehalococcoides strain DCMB5
DehaMB_2_0544*
YP_307395 Dehalococcoides strain CBDB1
DehaMB_1_0051*
YP_007484361 Dehalococcoides strain DCMB5
CCF77715 Uncultured Dehalococcoides sp.
Deha11a_2_0135**
YP_007485904 Dehalococcoides strain BTF08
YP_003330863 Dehalococcoides strain VS
YP_182243 Dehalococcoides strain 195
DehaMB_5_0010*
YP_001214759 Dehalococcoides strain BAV1
ABY28307 Uncultured Dehalococcoides sp. clone KS5
YP_007484365 Dehalococcoides strain DCMB5
DehaMB_1_0048*
DehaMB_1_0118*
YP_007485898 Dehalococcoides strain BTF08
YP_308563 Dehalococcoides strain CBDB1
Deha11a_2_0141**
YP_182236 Dehalococcoides strain 195
DehaMB_5_0004**
YP_180921 Dehalococcoides strain 195
DehaMB_2_0599**
YP_007483095 Dehalococcoides strain DCMB5
YP_003330780 Dehalococcoides strain VS
DehaMB_2_0523*
YP_181050 Dehalococcoides strain 195
DehaMB_1_0159*
ACF24861 Dehalococcoides strain MB
YP_308398 Dehalococcoides strain CBDB1
DehaMB_1_0007*
YP_003330848 Dehalococcoides strain VS
YP_308554 Dehalococcoides strain CBDB1
Deha11a_2_0150**
YP_007485888 Dehalococcoides strain BTF08
DehaMB_1_0129*
YP_003759128 Dehalogenimonas lykanthroporepellens BL-DC-9
YP_007484359 Dehalococcoides strain DCMB5
DehaMB_1_0053*
YP_307352 Dehalococcoides strain CBDB1
YP_001213642 Dehalococcoides strain BAV1
Deha11a_3_0062**
YP_003329669 Dehalococcoides strain VS
YP_180928 Dehalococcoides strain 195
DehaMB_2_0594*
Deha11a_4_0203**
YP_001214446 Dehalococcoides strain BAV1
YP_007483984 Dehalococcoides strain DCMB5
0.2

Supplementary Fig. S2. Neighbour joining tree of deduced amino acid sequences of *rdhA* genes in *Dhc* strains MB, 11a and top 2 hits of BLAST searches. RDases of strains MB and 11a are in bold and further denoted by a single asterisk identifies sequences from strain MB and two asterisks identify sequences from strain 11a. Numbers at nodes show bootstrap values of 1000 replicates. Branches without numbers had < 40% bootstrap values. The scale shows 20% amino acid differences. GenBank accession numbers of known RdhA comes before the taxonomic assignments.

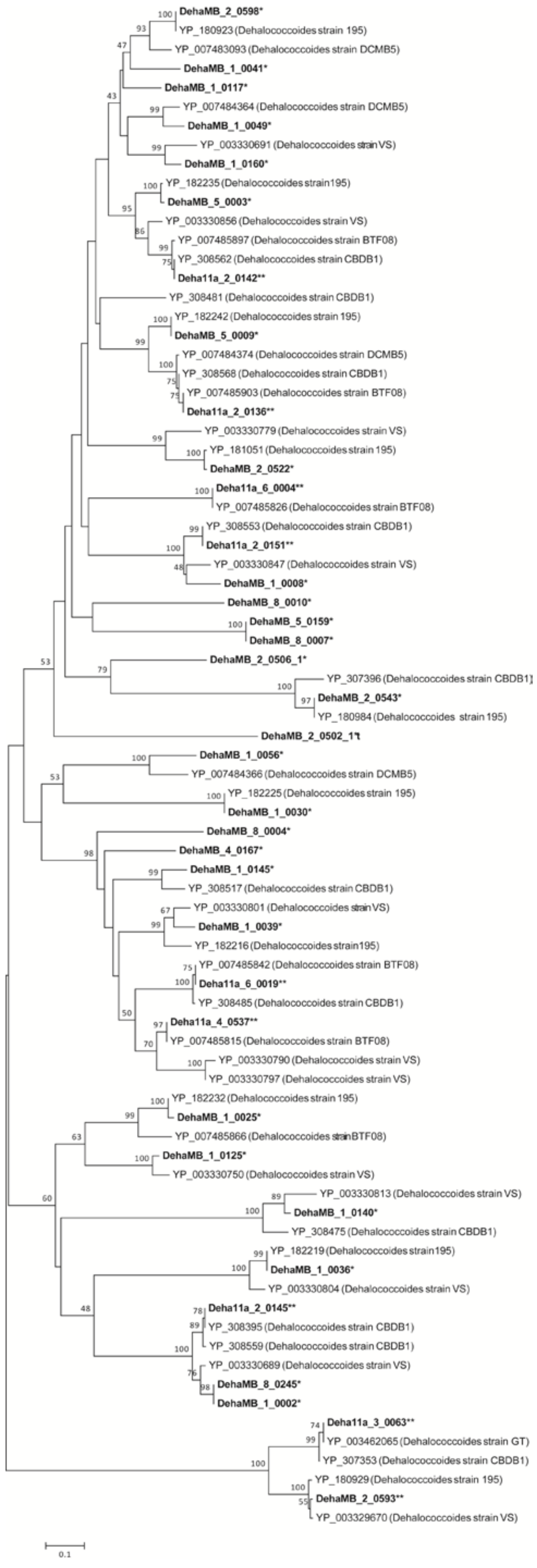
DehaMB_2_0598*
YP_180923 (Dehalococcoides strain 195)
YP_007483093 (Dehalococcoides strain DCMB5)
DehaMB_1_0041*
DehaMB_1_0117*
YP_007484364 (Dehalococcoides strain DCMB5)
DehaMB_1_0049*
YP_003330691 (Dehalococcoides strain VS)
DehaMB_1_0160*
YP_182235 (Dehalococcoides strain195)
DehaMB_5_0003*
YP_003330856 (Dehalococcoides strain VS)
YP_007485897 (Dehalococcoides strain BTF08)
YP_308562 (Dehalococcoides strain CBDB1)
Deha11a_2_0142**
YP_308481 (Dehalococcoides strain CBDB1)
YP_182242 (Dehalococcoides strain 195)
DehaMB_5_0009*
YP_007484374 (Dehalococcoides strain DCMB5)
YP_308568 (Dehalococcoides strain CBDB1)
YP_007485903 (Dehalococcoides strain BTF08)
Deha11a_2_0136**
YP_003330779 (Dehalococcoides strain VS)
YP_181051 (Dehalococcoides strain 195)
DehaMB_2_0522*
Deha11a_6_0004**
YP_007485826 (Dehalococcoides strain BTF08)
YP_308553 (Dehalococcoides strain CBDB1)
Deha11a_2_0151**
YP_003330847 (Dehalococcoides strain VS)
DehaMB_1_0008*
DehaMB_8_0010*
DehaMB_5_0159*
DehaMB_8_0007*
DehaMB_2_0506_1*
YP_307396 (Dehalococcoides strain CBDB1)
DehaMB_2_0543*
YP_180984 (Dehalococcoides strain 195)
DehaMB_2_0502_1*
DehaMB_1_0056*
YP_007484366 (Dehalococcoides strain DCMB5)
YP_182225 (Dehalococcoides strain 195)
DehaMB_1_0030*
DehaMB_8_0004*
DehaMB_4_0167*
DehaMB_1_0145*
YP_308517 (Dehalococcoides strain CBDB1)
YP_003330801 (Dehalococcoides strain VS)
DehaMB_1_0039*
YP_182216 (Dehalococcoides strain195)
YP_007485842 (Dehalococcoides strain BTF08)
Deha11a_6_0019**
YP_308485 (Dehalococcoides strain CBDB1)
Deha11a_4_0537**
YP_007485815 (Dehalococcoides strain BTF08)
YP_003330790 (Dehalococcoides strain VS)
YP_003330797 (Dehalococcoides strain VS)
YP_182232 (Dehalococcoides strain 195)
DehaMB_1_0025*
YP_007485866 (Dehalococcoides strain BTF08)
DehaMB_1_0125*
YP_003330750 (Dehalococcoides strain VS)
YP_003330813 (Dehalococcoides strain VS)
DehaMB_1_0140*
YP_308475 (Dehalococcoides strain CBDB1)
YP_182219 (Dehalococcoides strain195)
DehaMB_1_0036*
YP_003330804 (Dehalococcoides strain VS)
Deha11a_2_0145**
YP_308395 (Dehalococcoides strain CBDB1)
YP_308559 (Dehalococcoides strain CBDB1)
YP_003330689 (Dehalococcoides strain VS)
DehaMB_8_0245*
DehaMB_1_0002*
Deha11a_3_0063**
YP_003462065 (Dehalococcoides strain GT)
YP_307353 (Dehalococcoides strain CBDB1)
YP_180929 (Dehalococcoides strain 195)
DehaMB_2_0593**
YP_003329670 (Dehalococcoides strain VS)
0.1

Supplementary Fig. S3. A neighbour joining tree of deduced amino acid sequences of *rdhB* genes in *Dhc* strains MB, 11a and top 2 hits from nucleotide BLAST searches. RDases of strains MB and 11a are in bold and further denoted by a single asterisk identifies sequences from MB and two asterisks identify sequences from 11a. Numbers next to branches show bootstrap values of 1000 replicates. Branches without numbers had < 40% bootstrap values. The scale shows 10% amino acid differences. GenBank accession numbers of known RdhB comes before the taxonomic assignments.

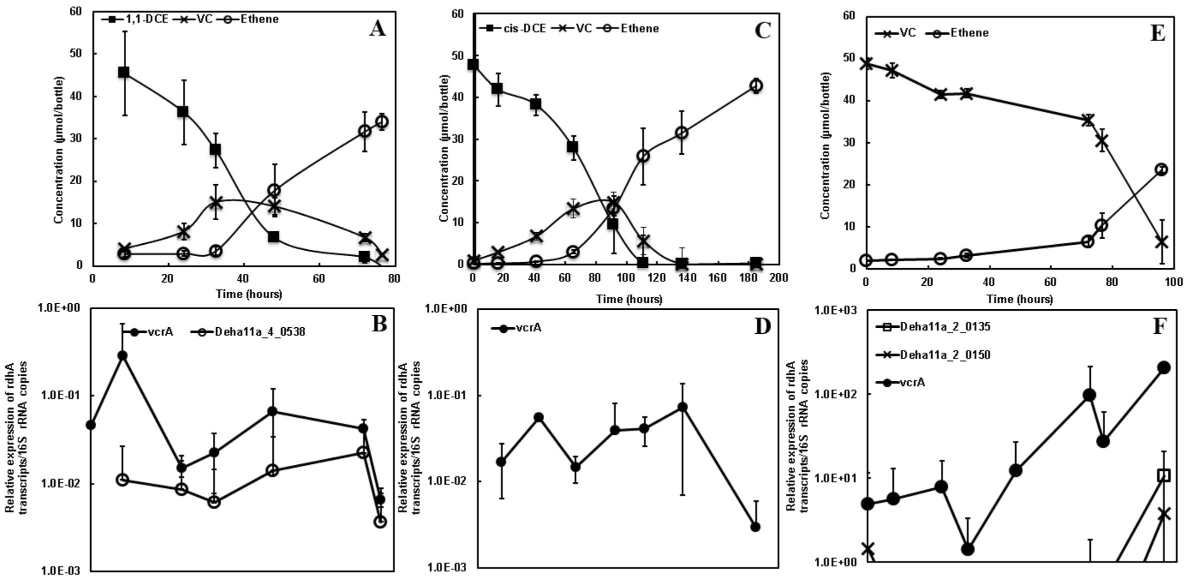

Supplementary Fig. S4. Dehalogenation profile and transcript expression of *rdhA* genes in strain 11a exposed to 1,1-DCE (A & B), cis-DCE (C & D), and VC (E & F). In the 1,1-DCE exposed cultures, *vcrA* transcripts were six-fold higher after 8 h than at 0 h of incubation during which VC appeared. In the cis-DCE exposed cultures, *vcrA* transcripts were an order of magnitude higher at 40 h than 0 h during which VC was produced. In the VC exposed cultures, *vcrA* transcripts were an order of magnitude higher at 100 h than 0 h during which ethene was produced. None of the other eight *rdhA* genes had the same transcript expression for the three organohalides. Error bars for *vcrA* genes and dehalogenation profiles are standard deviations of duplicate biological cultures.

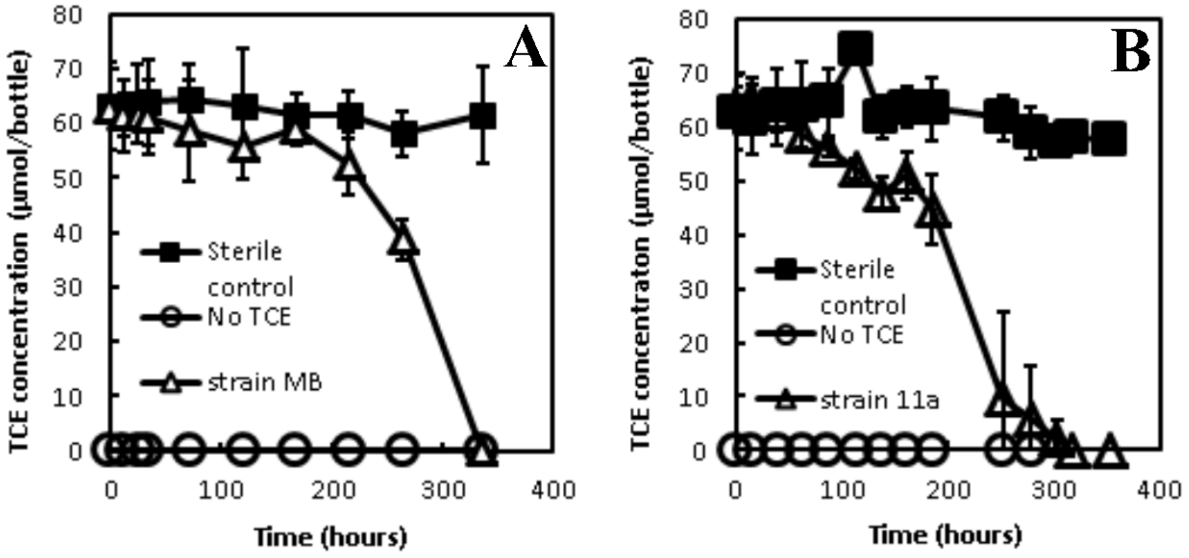

Supplementary Fig. S5. Complete TCE dehalogenation profile of strains MB (A) and 11a (B) along with sterile controls and cultures not amended with TCE. Error bars are standard deviation of biological triplicates.

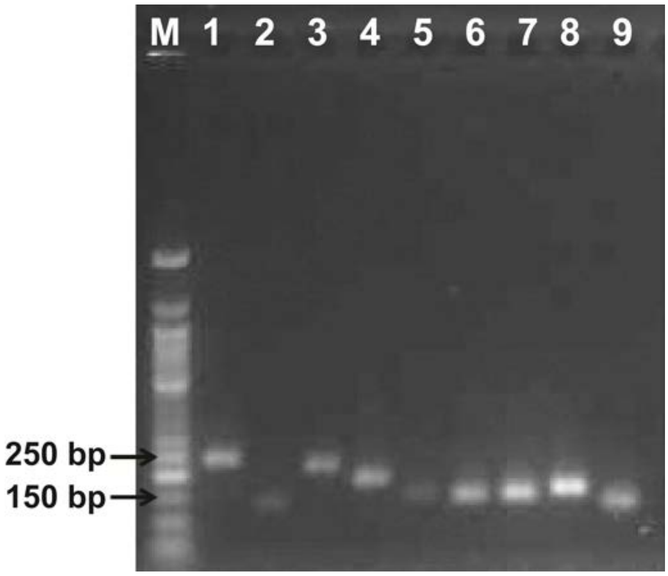

Supplementary Fig. S6. PCR products of 9 different *rdhA* genes of strain 11a DNA using specific primers. 1.8% TAE-agarose gel, lane M- 50 bp DNA ladder (New England Biolabs), lane 1- Deha11a_2_0135, lane 2- Deha11a_2_0141, lane 3- Deha11a_2_0144, lane 4-Deha11a_2_0150, lane 5- Deha11a_3_0062, lane 6- Deha11a_4_0203, lane 7- Deha11a_4_0536, lane 8-Deha11a_4_0538, lane 9-Deha11a_6_0005, the respective no template controls are in lanes 10 to 18.

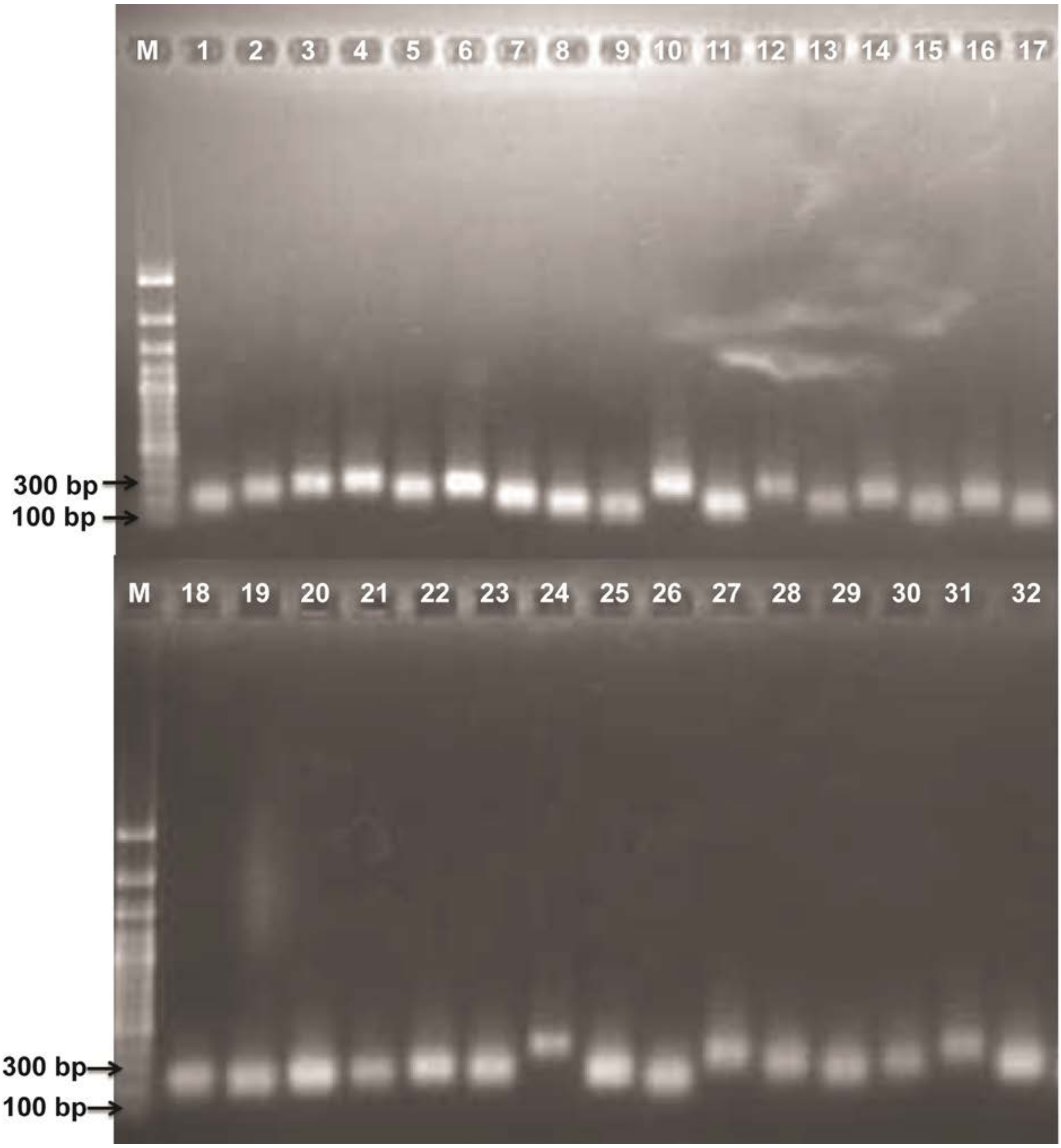

Supplementary Fig. S7. PCR products of 32 *rdhA* genes of strain MB DNA. The top gel is an image of a 1.5% TAE-agarose gel, lane M- GeneRuler 100 bp (Fermentas), lane 1-DehaMB_1_0007, lane 2-DehaMB_1_0024, lane 3-DehaMB_1_0029, lane 4-DehaMB_1_0035, lane 5-DehaMB_1_0038, lane 6-DehaMB_1_0040, lane 7-DehaMB_1_0044, lane 8-DehaMB_1_0048, lane 9-DehaMB_1_0051, lane 10-DehaMB_1_0053, lane 11-DehaMB_1_0055, lane 12-DehaMB_1_0059, lane 13-DehaMB_1_0118, lane 14-DehaMB_1_0119, lane 15-DehaMB_1_0124, lane 16-

DehaMB_1_0129, lane 17-DehaMB_1_0139. Bottom gel is an image of a 1.5% TAE agarose gel, lane 18-DehMB_1_0144, lane 19-DehaMB_1_0159, lane 20-DehaMB_2_0503, lane 21-DehaMB_2_0507, lane 22-DehaMB_2_0523, lane 23-DehaMB_2_0544, lane 24-DehaMB_2_0594, lane 25-DehaMB_2_0599, lane 26-DehaMB_2_0168, lane 27-DehaMB_5_0001, lane 28-DehaMB_5_0004, lane 29-DehaMB_5_0010, lane 30-DehaMB_5_157, lane 31-DehaMB_5_0160, lane 32-DehaMB_8_0011.

Supplementary Tables

Supplementary Table S1. Size of scaffolds of 11a and MB draft genomes.

Strains ID	Scaffold No.	Scaffold size (bp)
**11a**	1	1,239
	2	164,637
	3	627,157
	4	498,786
	5	13,378
	6	19,331
	Total:	1,324,528
**MB**	1	171,600
	2	689,467
	3	6,458
	4	337,793
	5	146,427
	6	1,415
	7	915
	8	225,587
	9	520
	10	1,276
	11	734
	Total:	1,582,192

Supplementary Methods

Nucleic acid extraction.

DNA used for genome sequencing were extracted from cells concentrated from 1 L of strains MB and 11a cultures by centrifugation at 8000× g for 20 mins in 50 mL conical tubes. High molecular weight genomic DNA was extracted using a Genomic-tip 100G (Qiagen GmbH, Hilden, Germany) as described in the manufacturer's protocol. For *rdhA* transcript expression study, RNA was extracted from 1.5 mL of cultures based on modifications to the RNeasy mini kit (Qiagen GmbH, Hilden, Germany) which included a DNA digestion step to remove DNA as previously described[1]. Cells were concentrated by centrifugation at 12000× *g* for 15 mins at 4 ^{o}C in 1.5 mL microtubes and all but 60 µL of the supernatant was aspirated. TRIzol® (Life Technologies, Carlsbad, CA, USA) of 150 µL was added to the samples before storing at -80 ^{o}C. Of 2×10^8 copies of luciferase RNA (Promega, Madison, WI, USA) were added to each sample prior to the first extraction step as a control for RNA extraction efficiency[2].

Reverse Transcription and Quantitative PCR.

Total RNA was converted to cDNA using the Sensiscript reverse transcription kit (Qiagen GmbH, Hilden, Germany) with random hexamers (Promega, Madison, WI, USA) as described in the manufacturer's protocol. Primers for each specific *rdhA* gene were designed to meet the criteria of the PCR conditions specified in the Sequence Manipulation Suite online primer stats program[3] and primers for the *Dhc* 16S rRNA gene (DhcF and DhcR)[4] and luciferase gene were designed as previously reported[2]. QPCR was performed on an ABI 7500 FAST real time PCR system (Applied Biosystems; Foster City, CA, USA). Supplementary Data 10 (online) contains the primer sequences and annealing temperatures used for qPCR. Standard curves for qPCR were generated using 10-fold serial dilutions of

respective *rdhA* genes, 16S rRNA gene or luciferase gene ligated onto pGEM-T vectors (Promega, Madison, WI, USA). These vectors were cloned into TOP10 chemically competent *Escherichia coli* cells (Invitrogen, Carlsbad, CA, USA) according to the manufacturer's protocol. Primer specificity was confirmed by PCR, showing bands of expected sizes using DNA extracted from strains 11a and MB (see Supplementary Fig. S6 and S7 online). QPCR was performed in a volume of 10 µL with technical duplicates. Total transcripts were normalized to RNA extraction efficiency using the measured quantity of luciferase RNA in each sample preparation (quantified luciferase transcript copies/expected luciferase transcript copies) before comparison with 16S rRNA gene. RDase transcripts from strain 11a grown in only with 1,1-DCE, *cis*-DCE or VC were normalised to 16S rRNA transcripts as previously described[5]. Absence of DNA contamination in the extracted mRNA was confirmed by direct PCR of RNA extracted from strains MB and 11a (data not shown)

Genome sequencing and Annotation.

The genomes of MB and 11a were sequenced on the Illumina GA-II genome analyser at the Beijing Genome Institute (BGI-Hong Kong). A paired-end sequencing method using 90 bp long reads and 200 bp insert library which yielded coverage depth of 110× per genome. The accession numbers for MB and 11a are stated in Table 1. Quality sequences were obtained using a quality score of 20 as the threshold[6]. A total of 1,888,074 and 1,689,890 high quality reads were generated for strains MB and 11a, respectively. To find the best assembly method, reads of strain 11a were assembled using Velvet version 1.2.03[7] and Ray version 20.0-rc8[8] and compared. To assemble the reads, the same k-mer sizes of 31, 41, 51, and 61 and contig size of > 500 bp were used for both strains in both assembly programs. The percentage of mapped reads over the raw reads using Ray and Velvet were 99.0% with N50 value of 498,785 bp and 98.8% with N50 value of 337,792 bp, respectively, indicating

that Ray had a slightly higher percentage of assembled reads and this program was subsequently used to assemble the raw reads of both strains SSPACE version 2.0[9] was used to obtain scaffolds, resulting in six scaffolds for strain 11a and 11 scaffolds for strain MB. The sizes of the scaffolds are stated in Supplementary Table S1 online).

Open reading frames (ORFs) were predicted using the AMIGene software[10] and the genomes of *D. mccartyi* strains 195 and BAV1 were used as reference genome models for strains MB and 11a, respectively. Functional annotations were assigned by screening predicted ORFs using the BLASTp in the BLAST+ package[11] to search against the NCBI non-redundant protein database[12] and UniRef90 databases[13]. Drafts genomes were further annotated using RAST[14]. The CDS nomenclatures for both strains are designated by name, scaffold number and CDS number (e.g. DehaMB_1_0001).

Genomic comparisons, phylogenetic trees construction, heatmap of non-core genes and clustered regularly interspaced short palindromic repeats.

To make graphical comparisons across genomes, the completed genomes of strains 195, BAV1, BTF08, DCMB5, CBDB1, GT, and VS were obtained from public databases, aligned and constructed using Circos[15]. Histograms of codon adaption index or codon usage bias (CUB) and G+C contents were calculated using CodonW version 1.4.4[16]. To compare the core and unique genes of genomes, blast2GO software[17] was used to cluster the genes into different cellular features based on annotations to the gene ontology (GO) database version 1.4[18].

Phylogenetic trees of deduced amino acid sequences of full-length RdhA and RdhB were aligned and constructed using MEGA version 5.0[19]. Partial sequences were not included in the trees. The phylogenetic trees were constructed using the neighbour joining method and 1,000 bootstrap replications were made to give confidence values at branches.

To obtain and compare orthologous, unique and non-core genes across *Dhc* strains, default parameters were used in the PanOCT software[20]. To make a heatmap of non-core genes, the Pheatmap version 0.7.7[21] package was run in *R*[22]. Clustered regularly interspaced short palindromic repeats (CRISPRs) were detected using the CRISPR finder program online[23].

References

1 Lee, P. K., Cheng, D., West, K. A., Alvarez-Cohen, L. & He, J. Isolation of two new Dehalococcoides mccartyi strains with dissimilar dechlorination functions and their characterization by comparative genomics via microarray analysis. *Environ. Microbiol.* **15**, 2293-2305 (2013).

2 Johnson, D. R., Lee, P. K., Holmes, V. F. & Alvarez-Cohen, L. An internal reference technique for accurately quantifying specific mRNAs by real-time PCR with application to the tceA reductive dehalogenase gene. *Appl. Environ. Microbiol.* **71**, 3866-3871 (2005).

3 Stothard, P. The sequence manipulation suite: JavaScript programs for analyzing and formatting protein and DNA sequences. *Biotechniques* **28**, 1102-1104 (2000).

4 Holmes, V. F., He, J., Lee, P. K. & Alvarez-Cohen, L. Discrimination of multiple *Dehalococcoides* strains in a trichloroethene enrichment by quantification of their reductive dehalogenase genes. *Appl. Environ. Microbiol.* **72**, 5877-5883, (2006).

5 Rowe, A. R., Heavner, G. L., Mansfeldt, C. B., Werner, J. J. & Richardson, R. E. Relating chloroethene respiration rates in Dehalococcoides to protein and mRNA biomarkers. *Environ. Sci. Technol.* **46**, 9388-9397 (2012).

6 Minoche, A. E., Dohm, J. C. & Himmelbauer, H. Evaluation of genomic high-throughput sequencing data generated on Illumina HiSeq and genome analyzer systems. *Genome Biol.* **12**, R112 (2011).

7 Zerbino, D. R. & Birney, E. Velvet: algorithms for de novo short read assembly using de Bruijn graphs. *Genome Res.* **18**, 821-829 (2008).

8 Boisvert, S., Raymond, F., Godzaridis, E., Laviolette, F. & Corbeil, J. Ray Meta: scalable de novo metagenome assembly and profiling. *Genome Biol.* **13**, R122, (2012).

9 Boetzer, M., Henkel, C. V., Jansen, H. J., Butler, D. & Pirovano, W. Scaffolding pre-assembled contigs using SSPACE. *Bioinformatics* **27**, 578-579, (2011).

10 Bocs, S., Cruveiller, S., Vallenet, D., Nuel, G. & Medigue, C. AMIGene: Annotation of MIcrobial Genes. *Nucleic Acids Res.* **31**, 3723-3726 (2003).

11 Camacho, C. *et al.* BLAST+: architecture and applications. *BMC bioinformatics* **10**, 421 (2009).

12 Tatusova, T., Ciufo, S., Fedorov, B., O'Neill, K. & Tolstoy, I. RefSeq microbial genomes database: new representation and annotation strategy. *Nucleic Acids Res.* **42**, D553-559 (2014).

13 Li, W. & Godzik, A. Cd-hit: a fast program for clustering and comparing large sets of protein or nucleotide sequences. *Bioinformatics* **22**, 1658-1659, (2006).

14 Aziz, R. K. *et al.* The RAST Server: rapid annotations using subsystems technology. *BMC genomics* **9**, 75 (2008).

15 Krzywinski, M. *et al.* Circos: an information aesthetic for comparative genomics. *Genome Res.* **19**, 1639-1645 (2009).

16 Peden, J. F. *Analysis of codon usage*, University of Nottingham, (1999).

17 Conesa, A. *et al.* Blast2GO: a universal tool for annotation, visualization and analysis in functional genomics research. *Bioinformatics* **21**, 3674-3676 (2005).

18 Ashburner, M. *et al.* Gene ontology: tool for the unification of biology. The Gene Ontology Consortium. *Nature genetics* **25**, 25-29 (2000).

19 Tamura, K. *et al.* MEGA5: molecular evolutionary genetics analysis using maximum likelihood, evolutionary distance, and maximum parsimony methods. *Molecular biology and evolution* **28**, 2731-2739 (2011).

20 Fouts, D. E., Brinkac, L., Beck, E., Inman, J. & Sutton, G. PanOCT: automated clustering of orthologs using conserved gene neighborhood for pan-genomic analysis of bacterial strains and closely related species. *Nucleic Acids Res* **40**, e172 (2012).

21 Kolde, R. pheatmap: pretty heatmaps. R package version 0.7.7. http://CRAN.R-project.org/package=pheatmap (2012).

22 R Core Team, R: A language and environment for statistical computing. (R Foundation for Statistical Computing, Vienna, Austria, 2010).

23 Grissa, I., Vergnaud, G. & Pourcel, C. The CRISPRdb database and tools to display CRISPRs and to generate dictionaries of spacers and repeats. *BMC bioinformatics* **8**, 172 (2007).

Supplementary Information

Supplementary Fig. S1. Neighbour joining tree of 16S rRNA genes of *Dhc* strains, numbers at nodes show bootstrap values using 1,000 replicates. Subgroup classification is indicated on the right. Scale bar shows 5% nucleotide base difference.

Supplementary Fig. S2. Neighbour joining tree of deduced amino acid sequences of *rdhA* genes in *Dhc* strains MB, 11a and top 2 hits of BLAST searches. RDases of strains MB and 11a are in bold and further denoted by a single asterisk identifies sequences from strain MB and two asterisks identify sequences from strain 11a. Numbers at nodes show bootstrap values of 1000 replicates. Branches without numbers had < 40% bootstrap values. The scale shows 20% amino acid differences. GenBank accession numbers of known RdhA comes before the taxonomic assignments.

Supplementary Fig. S3. A neighbour joining tree of deduced amino acid sequences of *rdhB* genes in *Dhc* strains MB, 11a and top 2 hits from nucleotide BLAST searches. RDases of strains MB and 11a are in bold and further denoted by a single asterisk identifies sequences from MB and two asterisks identify sequences from 11a. Numbers next to branches show bootstrap values of 1000 replicates. Branches without numbers had < 40% bootstrap values. The scale shows 10% amino acid differences. GenBank accession numbers of known RdhB comes before the taxonomic assignments.

Supplementary Fig. S4. Dehalogenation profile and transcript expression of *rdhA* genes in strain 11a exposed to 1,1-DCE (A & B), cis-DCE (C & D), and VC (E & F). In the 1,1-DCE exposed cultures, *vcrA* transcripts were six-fold higher after 8 h than at 0 h of incubation during which VC appeared. In the cis-DCE exposed cultures, *vcrA* transcripts were an order of magnitude higher at 40 h than 0 h during which VC was produced. In the VC exposed cultures, *vcrA* transcripts were an order of magnitude higher at 100 h than 0 h during which ethene was produced. None of the other eight *rdhA* genes had the same

transcript expression for the three organohalides. Error bars for *vcrA* genes and dehalogenation profiles are standard deviations of duplicate biological cultures.

Supplementary Fig. S5. Complete TCE dehalogenation profile of strains MB (A) and 11a (B) along with sterile controls and cultures not amended with TCE. Error bars are standard deviation of biological triplicates.

Supplementary Fig. S6. PCR products of 9 different *rdhA* genes of strain 11a DNA using specific primers. 1.8% TAE-agarose gel, lane M- 50 bp DNA ladder (New England Biolabs), lane 1- Deha11a_2_0135, lane 2- Deha11a_2_0141, lane 3- Deha11a_2_0144, lane 4-Deha11a_2_0150, lane 5- Deha11a_3_0062, lane 6- Deha11a_4_0203, lane 7- Deha11a_4_0536, lane 8-Deha11a_4_0538, lane 9-Deha11a_6_0005, the respective no template controls are in lanes 10 to 18.

Supplementary Fig. S7. PCR products of 32 *rdhA* genes of strain MB DNA. The top gel is an image of a 1.5% TAE-agarose gel, lane M- GeneRuler 100 bp (Fermentas), lane 1-DehaMB_1_0007, lane 2-DehaMB_1_0024, lane 3-DehaMB_1_0029, lane 4-DehaMB_1_0035, lane 5-DehaMB_1_0038, lane 6-DehaMB_1_0040, lane 7-DehaMB_1_0044, lane 8-DehaMB_1_0048, lane 9-DehaMB_1_0051, lane 10-DehaMB_1_0053, lane 11-DehaMB_1_0055, lane 12-DehaMB_1_0059, lane 13-DehaMB_1_0118, lane 14-DehaMB_1_0119, lane 15-DehaMB_1_0124, lane 16-DehaMB_1_0129, lane 17-DehaMB_1_0139. Bottom gel is an image of a 1.5% TAE agarose gel, lane 18-DehMB_1_0144, lane 19-DehaMB_1_0159, lane 20-DehaMB_2_0503, lane 21-DehaMB_2_0507, lane 22-DehaMB_2_0523, lane 23-DehaMB_2_0544, lane 24-DehaMB_2_0594, lane 25-DehaMB_2_0599, lane 26-DehaMB_2_0168, lane 27-DehaMB_5_0001, lane 28-DehaMB_5_0004, lane 29-DehaMB_5_0010, lane 30-DehaMB_5_157, lane 31-DehaMB_5_0160, lane 32-DehaMB_8_0011.

Supplementary Table S1. Size of scaffolds of 11a and MB draft genomes.

Supplementary Methods. DNA and RNA extractions; Reverse Transcription and Quantitative PCR; Genome sequencing and Annotation; Genomic comparisons, phylogenetic trees construction, heatmap of non-core genes and clustered regularly interspaced short palindromic repeats.

Supplementary Data 1. Genomic summary of 9 Dehalococcoides strains and a single Dehalogenimonas strain.

Supplementary Data 2. Protein coding sequence (CDS) only found in strain MB among seven Dehalococcoides strains 195, BAV1, BTF08, CBDB1, DCMB5, GT, VS.

Supplementary Data 3. Protein coding sequence (CDS) only found in strain 11a among seven Dehalococcoides strains 195, BAV1, BTF08, CBDB1, DCMB5, GT, VS.

Supplementary Data 4. Protein coding sequence (CDS) within high plasticity regions (HPR) of strain MB arranged in the order of alignment to strain 195.

Supplementary Data 5. Protein coding sequence (CDS) within high plasticity regions (HPR) of strain 11a arranged in the order of alignment to strain BAV1.

Supplementary Data 6. Annotations of rdhA genes using UniRef90 and NCBI.

Supplementary Data 7. Annotations of rdhB genes using UniRef90 and NCBI.

Supplementary Data 8. Protein coding sequence (CDS) of strain MB annotated using UniRef90.

Supplementary Data 9. Protein coding sequence (CDS) of strain 11a annotated using UniRef90.

Supplementary Data 10. qPCR primers and annealing temperature.

Supplementary Data 11. Protein coding sequences (CDS) related to the corrinoid synthesis pathway of strain 11a.

Supplementary Data 12. Protein coding sequences (CDS) related to the corrinoid synthesis pathway of strain MB.
